# Diagnostic tools for soil-transmitted helminths control and elimination programs: A pathway for diagnostic product development

**DOI:** 10.1371/journal.pntd.0006213

**Published:** 2018-03-01

**Authors:** Mark D. Lim, Simon J. Brooker, Vicente Y. Belizario, Françoise Gay-Andrieu, John Gilleard, Bruno Levecke, Lisette van Lieshout, Graham F. Medley, Zeleke Mekonnen, Greg Mirams, Sammy M. Njenga, Maurice R. Odiere, James W. Rudge, Lieven Stuyver, Jozef Vercruysse, Johnny Vlaminck, Judd L. Walson, the Annecy STH diagnostic experts group

**Affiliations:** 1 Global Health Division, The Bill & Melinda Gates Foundation, Seattle, United States of America; 2 College of Public Health, University of Philippines, Manila, Philippines; 3 bioMérieux, Marcy l’Etoile, France; 4 Faculty of Veterinary Medicine, University of Calgary, Calgary, Canada; 5 Faculty of Veterinary Medicine, Gent University, Merelbeke, Belgium; 6 Department of Parasitology, Leiden University Medical Center, Leiden, the Netherlands; 7 Department of Global Health and Development, London School of Hygiene and Tropical Medicine, London, United Kingdom; 8 Jimma University Institute of Health, Jimma, Ethiopia; 9 Techion Group Ltd, Dunedin, New Zealand; 10 Kenya Medical Research Institute, Nairobi, Kenya; 11 Centre for Global Health Research, Kenya Medical Research Institute, Kisumu, Kenya; 12 Janssen Diagnostics, Beerse, Belgium; 13 Departments of Global Health, Medicine (Infectious Disease), Pediatrics and Epidemiology, University of Washington, United States of America; 14 Natural History Museum, London, United Kingdom; Miyazaki Daigaku Igakubu Daigakuin Ikagaku Kangogaku Kenkyuka, JAPAN

## Introduction

The 2020 Roadmap goals endorsed by the World Health Organization (WHO) for soil-transmitted helminths (STHs) (*Ascaris lumbricoides*, *Ancylostoma duodenale*, *Necator americanus*, *Trichuris trichiura*) are focused on mass drug administration (MDA) of anthelmintics to control morbidity associated with moderate- and heavy-intensity infection [1]. As the STH community approaches the 75% coverage target for preschool- and school-aged children, there is increasing interest in exploring post-2020 goals that transition from simply monitoring program coverage to strengthened monitoring of a program’s impact on transmission of infection and determining whether enhanced MDA can break STH transmission with minimal risk of recrudescence [2–4].

Diagnostics play a critical role in guiding both the deployment of existing STH program resources and the implementation and evaluation of STH intervention strategies. Currently used coproscopic methods to detect and quantify STH-specific eggs, such as the Kato-Katz method, have practical advantages; test kits are inexpensive and relatively easy to perform in low-resourced field settings. They also have significant disadvantages, including moderate labor costs, lower than optimal sensitivity, and poor reproducibility in most program settings. Several academic and small-business efforts continue to develop tools with improved diagnostic performance [5–7]. However, an objective assessment on the value proposition offered by these tools has been complicated, as the diagnostic needs of a multiphased STH program have not been defined [8].

This report shares a user-centered framework developed by a diverse group of key opinion leaders convened over the past year by the Bill & Melinda Gates Foundation to define circumstances in which population-level diagnostic data could guide an STH program manager’s decision to transition a program to the next phase. The use-cases and companion target product profiles (TPPs) are intended to provide the community with a pathway for the research, development, evaluation, and implementation of diagnostic tools designed for STH programs. This framework can also be used to prioritize research or product development resources based on immediate and anticipated program needs.

### Current landscape of STH program diagnostics

The number of adult STH worms harbored by an individual determines both their risk of morbidity and contribution to overall transmission [9]. Worm expulsion studies required to quantify worm burden have suboptimal accuracy, are laborious, and are rarely done. Thus, STH programs employ indirect methods to infer worm burden, such as microscopy-based technologies for visual identification and quantification of STH-specific eggs from a stool sample. WHO recommends the use of the Kato-Katz method, a low-cost, simple, and standardized tool that provides sufficient sensitivity for morbidity control programs aiming to reduce prevalence of moderate- and heavy-intensity infections to less than 1% [10]. A key limitation of the method is its suboptimal sensitivity, particularly in low transmission settings where egg counts are typically low [11]. Alternatives to Kato-Katz include the Mini-FLOTAC and McMaster methods, although these tools lack WHO recommendations for programs and thus have been limited to research use [5, 11–13]. Recent technology development efforts have also focused on improved analytical sensitivity, such as molecular assays [6, 14–17] and enhanced visualization of helminth eggs [7, 18]. Another early area of investigation includes serological and urine-based measurements [19, 20]. However, all these methods potentially incur additional costs-per-test and resource requirements for STH programs that need to be considered, relative to the benefits of enhanced efficiency and accuracy [21]. Table 1 highlights other opportunities to improve coproscopic methods.

**Table 1:** Limitations and opportunities for improving coproscopy, with the Kato-Katz method as a predicate technique.

Limitations • Low sensitivity for very low-intensity infections • Variable test results impact prevalence measurements, particularly if eggs are highly clustered or in low abundance • Intra-individual variation in egg excretion during the day and between consecutive days • Operator-based variability in test results • Exposure of operator to infectious agents in stool • Need to process stool samples quickly after collection, particularly for hookworm analysis	Opportunities for improvement • Integrated quality control/quality assurance for preparation (homogenization) and analysis of stool samples • Increased throughput • Electronic connectivity, test results accessible for remote interpretation

### Starting from the end: STH diagnostic use-cases and TPPs

Diagnostics are required at different decision points in STH programs, ranging from mapping endemic geographies to monitoring and evaluation, assessing whether MDA can be stopped, and post-MDA surveillance [22]. Use-cases depict the link between a specific program decision to the interpretation of a diagnostic test result, regardless of the technology or method used to make the measurement [8]. A group of key opinion leaders represented the voice of the diagnostic user by describing and predicting scenarios faced by STH programs, creating a series of problem statements and decisions that each can be addressed by a hypothetical diagnostic. Each solution is further detailed in a TPP as a list of technical characteristics, such as type of measurement and implementation requirements.

One practical use of a TPP is to provide an objective framework for evaluating existing technologies and innovations to determine opportunities for product development (Table 2). The breakdown of an STH program into diagnostic use-cases also ensures that research and product development resources are aligned with program time lines by considering global progress of STH programs and goals (controlling morbidity, interruption of transmission), maturity of technology landscape, and time lines when technologies will be needed. This framework is not intended to prevent the development of a single technology that addresses multiple use-cases; however, a platform must meet the requirements described in each of the various TPPs.

**Table 2:** Planning processes for product development.

Output	Objective	Stakeholders responsible for definition
Use-cases	• Requires understanding of program workflow; infrastructure; resources to identify needs, preferences, limitations for implementation • Define link between phase of program and criteria, with implications of diagnostics-based decision • Frame epidemiological/biological characteristics • Identify stakeholders (data users, test implementers, policy, payors)	• Programs/implementers • WHO STH program guidelines or recommendations With input from: • Research (laboratory, epidemiology, field)
TPP	• Requires defined use-case • Define “must-have” criteria for a diagnostic to meet program needs for each use-case • Assess feasibility of meeting TPP requirements via landscape of existing research/methods/technologies/available patient specimens • Define evaluation criteria and methods for assessing new products and regulatory pathway • Account for cost-effectiveness, scalability, manufacturability	• Programs/implementers • Research • Product development With input from: • WHO STH program guidelines or recommendations • Regulatory
Product development pathway	• Requires use-case and TPP • Define regulatory pathway • Define requirements in accordance with existing or new STH program policies/guidelines/recommendations • Define pathway for translating existing reagents/methods into prototype • Define verification criteria that prototype meets TPP to lock/freeze design • Define validation criteria to evaluate adherence to TPP in actual operating conditions • Define product launch plan • Define payors/donors for program’s diagnostic infrastructure • Define user-support, quality assurance, monitoring, supply chain requirements • Define manufacturing and distribution plans	• Research • Product development • Regulatory • WHO STH program guidelines or recommendations With input from: • Country programs • Representatives from Ministries of Health • Program donors • Implementing nongovernment organizations

Abbreviations: STH, soil-transmitted helminth; TPP, target product profile.

Previous works by Solomon et al. [22] and Hawkins et al. [23] have provided high-level TPPs. This report builds on these efforts by providing a more comprehensive framework that links program decision points to detailed use-cases and TPPs. The diagnostic end user is the STH program manager who requires a population-wide diagnostic assessment to determine the transition of a program to the next planned phase. This introduces a unique challenge for developing diagnostic TPPs for population-based intervention programs, as performance requirements for individual-level assays are in context of decisions informed by population-level indicators.

**Fig 1:**
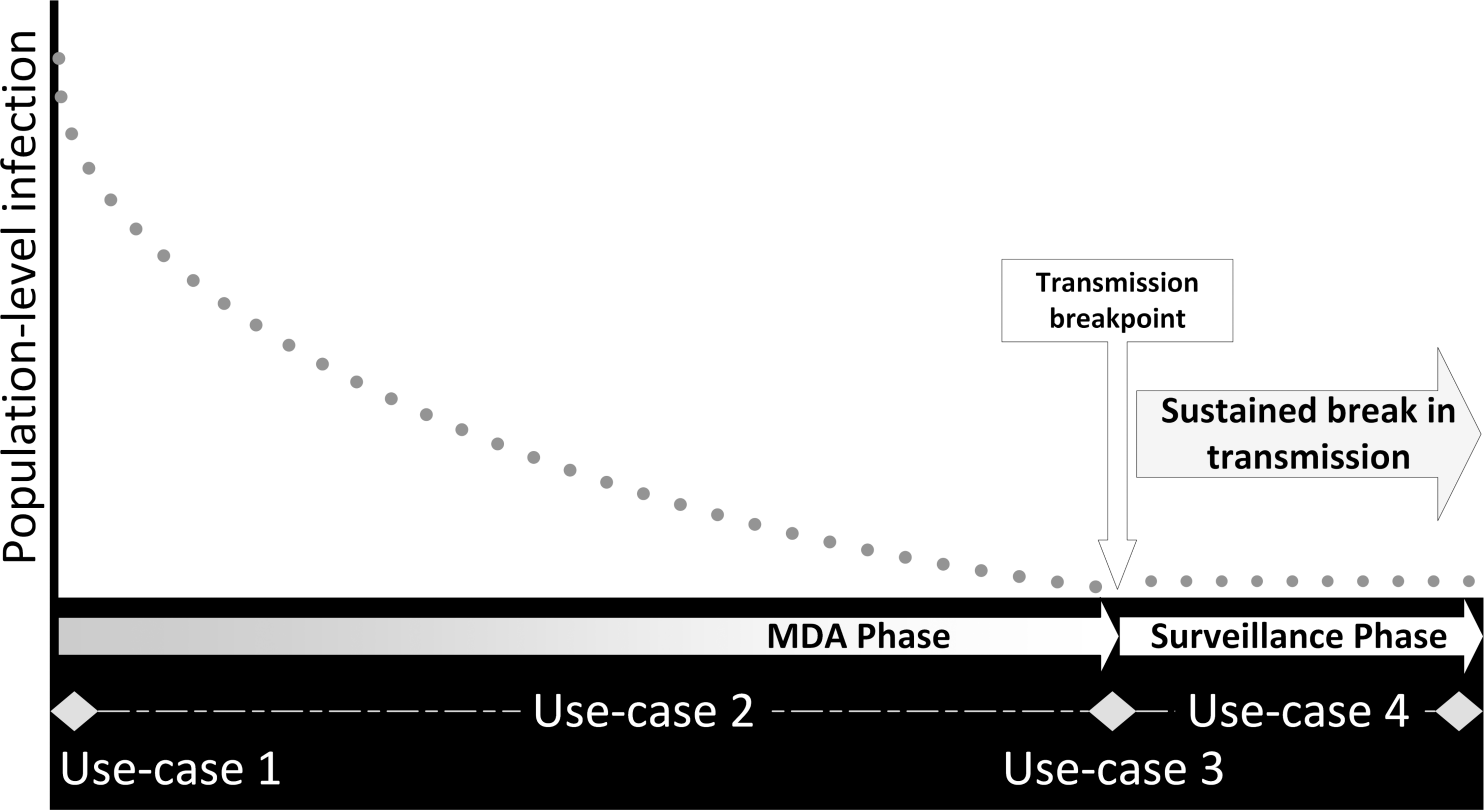
Hypothetical prevalence curve (dots) for a successful STH elimination program, overlaid with program phase (diamond for transition points) and diagnostic use-cases. MDA, mass drug administration; STH, soil-transmitted helminth.

**Fig 2:**
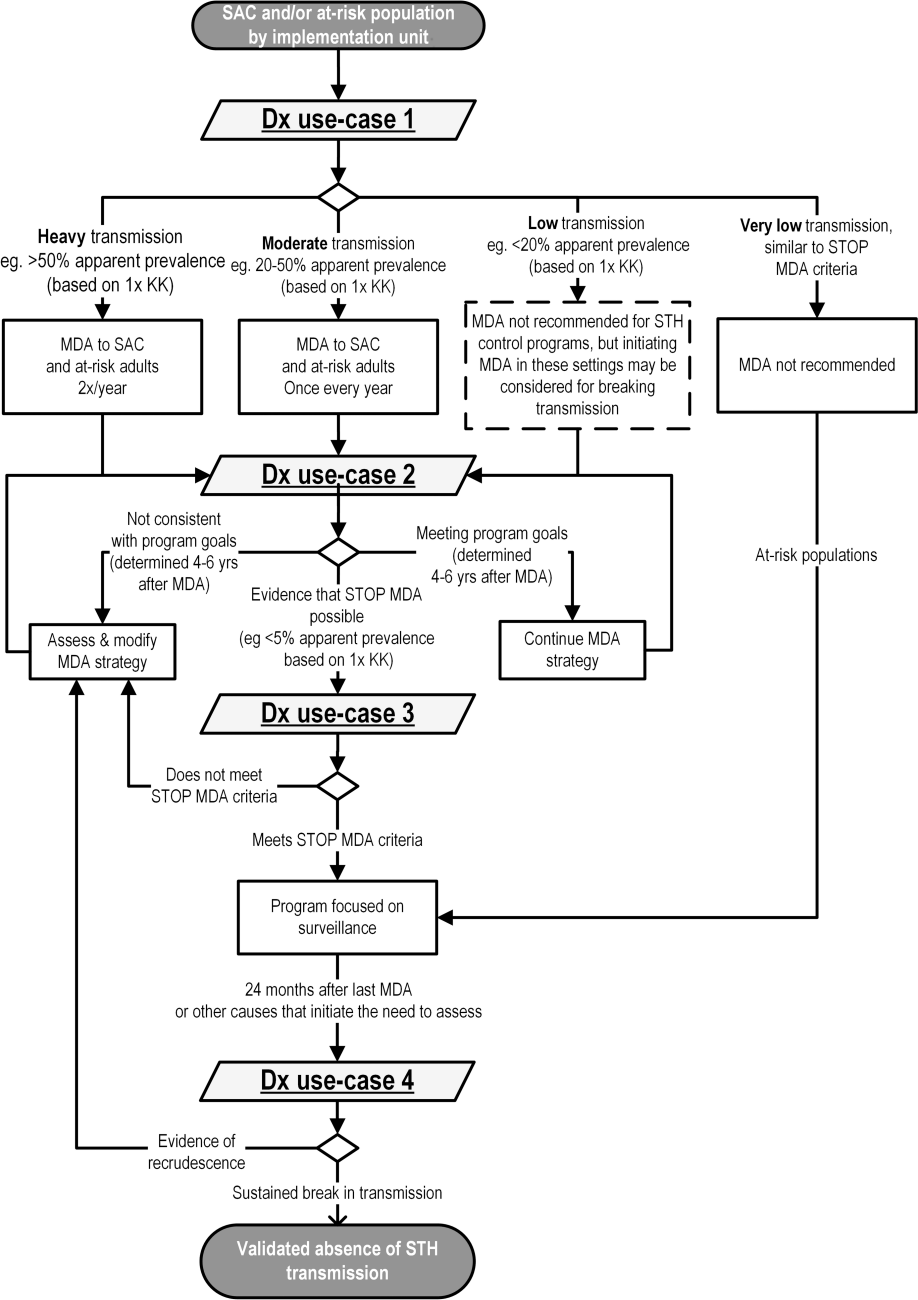
Diagnostic use-cases, described by program decision algorithm. Dashed box indicates a decision not described under current WHO guidelines for controlling STH morbidity but that may be important for a program aiming to eliminate transmission of STH. Dx, diagnostic; KK, Kato-Katz; MDA, mass drug administration; SAC, school-aged children; STH, soil-transmitted helminth.

Following previous work [22], four broad decision points were used to categorize each use-case against a hypothetical reduction in population-level infection resulting from program intervention, as shown in Fig 1. Embedded within this illustration is a spectrum of program decisions to initiate, continue, suspend, or transition to the next planned program phase, in the context of a program’s goal of morbidity control or elimination of transmission. Those decisions that are hypothetically guided by diagnostic test results are described in the algorithm shown in Fig 2 and form the basis of the use-case categories:

- **use-case #1.** Determine STH transmission and identify type of MDA,
- **use-case #2**. Assess progress against program goals,
- **use-case #3**. Confirm a decision to stop intervention and transition to surveillance,
- **use-case #4**. Verify sustained break in transmission.

An additional level of detail is provided in the spreadsheet within the supplementary materials of this article (S1 File).

Several factors were considered in prioritizing a diagnostic that confirms a break in transmission (use-case #3). There is strong interest in leveraging the successes of increased MDA coverage to further reduce STH transmission beyond the level of morbidity control toward interruption of transmission [2, 3]. Achievement of this goal would allow programs to stop regular MDA with minimal risk of recrudescence, but there currently lacks a reliable tool to confirm this end point. However, as discussed later, evidence from research is needed to develop a rigorous TPP for such a diagnostic. On the shorter term, there may be opportunities to strengthen STH programs by providing access to technologies that are superior to the Kato-Katz method (Table 1) for monitoring impact on transmission (use-case #2) [24].

The development of a TPP for use-case #4 was felt to be premature and not to be pursued until evidence demonstrates that a strategy for sustained interruption of transmission is feasible and can be scaled for STH programs. The biomarker landscape that would meet this context of use is also at its early stages, and resources required to implement the scale and coverage for this type of surveillance infrastructure need to be further defined [25].

Across all use-cases is an option to integrate and leverage the resources of other public health programs. For instance, many STH programs are integrated with schistosomiasis control efforts due to co-endemicity [8, 26], with the Kato-Katz method also able to detect infection by *Schistosoma mansoni* and *S*. *japonicum*. Non–stool-based biomarkers may provide opportunities for simultaneous detection of infection by *S*. *haematobium*. Post-elimination surveillance would likely leverage multiple disease surveillance programs through centralized laboratory analysis.

### Use-case #1—Initiate and determine type of MDA

The assay described in this use-case provides results that identify populations that warrant MDA and determines the frequency of MDA and the frequency of future monitoring. These decisions are currently based on measurements of two parasitological indicators in school-aged children: overall prevalence of any STH infection and proportion of individuals harboring an infection of moderate to heavy intensity [27].

The first indicator relies on aggregated results from individual-level diagnostic tests to determine whether the prevalence of STH infection is below 20%, exceeds 20%, or is 50% and above. These thresholds have been defined by WHO and are based on fecal egg counts (FECs) derived from a Kato-Katz measurement to guide an STH control program in not providing MDA or providing annual or biannual MDA. Because these programs are currently focused on controlling morbidity from moderate- to heavy-intensity infection, there are no recommendations for prevalence less than 20%, nor is the Kato-Katz method suitable for measuring low FEC. However, a “low transmission” category was included in the population stratification (Fig 2) in the hypothetical event that STH elimination programs would be initiated in lower transmission settings and that a tool that detects lighter infections would be available.

To meet the needs of the second indicator, a test provides individual-level quantitative results that are combined to estimate the proportion harboring moderate to heavy intensities of infection by any STH, thresholds currently based on species-specific FEC (Table 3, [1]). These test results also provide a baseline measurement for monitoring the impact of an intervention, as described in latter use-cases.

**Table 3:** Classes of intensity, based on Kato-Katz measurements [1].

STH	Individual intensity of infection (in eggs per gram of stool)		
	Light	Moderate	Heavy
*Ascaris lumbricoides*	1–4,999	5,000–49,999	≥50,000
*Trichuris trichiura*	1–999	1,000–9,999	≥10,000
Hookworms	1–1,999	2,000–3,999	≥4,000

**Abbreviation:** STH, soil-transmitted helminth.

### Use-case #2—Assess progress against program goals

This use-case applies to STH programs that have initiated intervention and seek to evaluate progress in reducing prevalence and intensity of infection [28]. By comparing population-level results from previous or baseline measurements, programs that meet their milestones would continue the intervention strategy as planned. However, an under-performing program would conduct additional evaluations to determine potential causes, such as assessments of population migration, environment, workforce, drug quality, treatment adherence, and anthelmintic drug resistance.

Decisions made from these quantitative tests are dependent on the type of program and this use-case was divided into two. Morbidity control programs are initiated in moderate- to high-prevalence settings and rely on two indicators, overall prevalence of infection by any STH and the proportion of individuals with moderate and heavy infections (e.g., use-case #2A, as described in S1 File). An STH program targeting interruption of transmission would initiate or continue efforts in lower transmission settings and would be focused solely on monitoring the reduction of overall prevalence (e.g., use-case #2B), because it is unlikely that there will be any moderately to heavily infected individuals [29].

The Kato-Katz method provides sufficiently reliable analytical data to meet use-case #2A and is suitable for morbidity control programs focused on moderately to heavily infected individuals, but improvements to FEC measurements that address reproducibility and throughput challenges (Table 1) would enhance program efficiency as well as create opportunities for programs to proceed beyond morbidity control [30, 31]. Technologies that meet the needs of use-case #2B can also be used in moderate and high transmission settings by STH control programs but offer greater value in lower transmission settings, where the Kato-Katz method fails to provide reliable data. For practical considerations, a preferred technology would be a platform that addresses multiple use-cases by meeting the requirements described in each of the associated TPPs.

### Use-case #3—Confirm a decision to stop intervention and transition to post-MDA surveillance

This use-case applies to programs aiming to interrupt transmission of any STH in low- to very-low- prevalence settings. In this circumstance, program progress and other transmission measurements would lead a program manager to initiate a test that confirms that a program can stop MDA and/or other population-directed interventions. Diagnostic results that confirm a break in transmission with minimal risk of recrudescence would transition program goals from active intervention to surveillance for recrudescence (use-case #4) or, in the event of discordant results, would initiate additional assessments.

The low- to very-low-prevalence threshold that describes the transmission breakpoint is species specific and has yet to be established, although it can be approximated through mathematical and animal models [32]. Based on studies with *A*. *lumbricoides*, worm dynamics behave differently at low worm burdens compared to high burdens, such that the number of fertile eggs no longer have a linear relationship to worm burden, making any individual-level coproscopic measurement unreliable for determining very light intensities of infection relevant to the population-level transmission breakpoint [33]. Nonmicroscopy biomarkers with a linear relationship to low worm burden, detectable within a dynamic range relevant to the transmission breakpoint, are needed. Table 4 provides a high-level overview of desired characteristics for any biomarker meeting this use-case.

**Table 4:** General desired characteristics of use-case #3 and #4 biomarkers.

• Biomarker measurement correlates to active infection by specific species-level STH • Biomarker clears within 1 year of last prescribed intervention, in absence of reinfection • Specific for each STH, no cross-reactivity with other pathogens • Detected in populations residing in geographies with less than 2% prevalence of any STH infection • Detected in infected individuals who are Kato-Katz negative • Sufficient abundance in readily accessible body fluid (nonstool) • Detection maximizes cost-efficiencies (e.g., amenable to pooling, simplified collection and shipment, testing in young children born after presumed transmission breakage) • Easily translatable to accessible diagnostic platforms

**Abbreviation:** STH, soil-transmitted helminth.

### Use-case #4—Verify sustained break in transmission

This use-case applies to programs that have successfully interrupted STH transmission (use-case #3) and seek to verify sustained elimination of transmission [34] or have reason to believe that there may be a risk of recrudescence. A program would investigate the potential causes of unexpected results if qualitative test results indicate ongoing transmission. Ideally, the test detects other diseases under surveillance and requires a specimen that is easier to collect than stool, such as urine, saliva, or blood, to encourage participation in screening events, with analysis performed in a centralized laboratory.

The aspirational list of biomarker characteristics described in Table 4 are similar for use-cases #3 and #4, and stricter definitions warrant further discussion. Similar tests for other diseases have relied on detecting host-response antibodies of infection [35], a class of biomarker convenient for integrated surveillance. These biomarkers of host response should ideally be specific for active infection, not exposure. Alternatively, exposure-based biomarkers could be measured within indicator subpopulations born after transmission has been broken, as these groups should not have been exposed to STH infection in geographies remaining absent of transmission [36]. Although species-specific detection is listed in Table 4, it remains unclear if a pan-STH biomarker would suffice for this use-case.

### Use-cases to TPPs

The TPP describes technical performance and implementation requirements for an assay to meet the decision needs of a program’s specific use-case. Each requirement is defined in a TPP as minimal or optimal criteria, reflecting a consensus of accepted compromises. To reduce risks and time lines for developing a product and accelerating adoption, criteria should consider the capacity, resources, and diagnostic workflows of STH programs in the context of existing research, methodology, and technology landscape.

Requirements for technology performance and implementation are linked and criteria consider trade-offs for supporting a new test within an existing diagnostic system versus costs for adapting or creating infrastructure. Implementation considerations include: survey design (population targeted for testing, sampling size), available workforce, workflow (specimen collection and transportation, sample preparation and analysis), throughput and turnaround time for test results, data requirements, and criteria for reimbursing test costs. Other considerations include external quality assurance requirements and regulatory pathway as well as program recommendations, policies, and guidelines.

Because diagnostics only approximate a true state of infection, mathematical models can be used to further inform performance requirements by estimating the impact of different levels of uncertainty on the accuracy of program decision-making and ultimately on health outcomes [37]. Models can also provide a health economics framework to justify performance requirements by weighing the predicted health outcomes against costs incurred by a program to conduct a survey as well as resources deployed in the event of an incorrect decision [3].

Minimum criteria describe performance characteristics that must be achieved for a test to be used by most STH programs. Optimal criteria describe attributes that expand the value of the assay but would not be required by most STH programs. Simply, minimum criteria describe “must-have” requirements, whereas “nice-to-have” options are listed as optimal, details that guide product development and evaluation priorities as well as resource allocation. In addition to setting targets for technology development, these requirements are also criteria for clinical and field trials and should consider availability and access to patient specimens from geographies representing the epidemiological and individual context of intended-use populations. These specimens must naturally represent the diversity and range of biomarkers to validate the performance claims of a prototype assay, with appropriate analytical and clinical benchmarks. Excessive technical complexity beyond actual program needs should be avoided, as each claim needs to be validated with available patient samples.

For example, qualitative yes/no results may be a sufficient level of detail that most programs require from a test result, such as presence or absence of transmission within a population (e.g., use-case #3). In this instance, the minimum requirement listed in a TPP would be a qualitative test result, for interpretation by a program manager. It is important to differentiate the presentation of a test result from the method of analysis, as this qualitative output could be derived from the quantitative analysis of aggregated individual-level data or pooled specimens. If some programs have the resources and capacity to also act on test results that provide species-specific intensities of infection, then quantitation could be listed as optimal criteria. However, validating that a test reliably provides quantitative test results for each STH species also requires access to statistically powered quantities of accessible patient specimens that contain the natural dynamic range of intensities for each STH.

As mentioned earlier, the use-case for monitoring program impact was divided into two similar use-cases, #2A and #2B, to address programs that intend to reduce transmission beyond morbidity control (S1 File). However, a TPP for use-case #2B was not developed, as a lower limit of detection (LOD) would approximate the transmission breakpoint, a species-specific indicator that requires further definition. At these lower transmission settings, a program manager might be solely interested in prevalence, unlike morbidity control programs, in which intensities of infection are an additional program metric.

Two TPPs were developed and finalized by this group of STH stakeholders. Use-cases #1 and #2A were combined because there is little demand for a diagnostic dedicated to use-case #1, with the current pace of coverage by STH programs (S2 File). Diagnostic tools that address both use-cases would likely be similar, given the current landscape of coproscopy technologies. There was agreement on current WHO recommendations for using the Kato-Katz method in morbidity control programs, but this technique would not meet all requirements described in this new TPP. With an intent to strengthen a program’s ability to monitor impact, new diagnostic products must satisfy all minimum requirements described in this joint TPP.

The second TPP described a tool to confirm a sustained break in transmission, a use-case that only requires a qualitative test result (S3 File). Conceptually, a platform that meets the needs of use-case #3 might also satisfy use-case #1–#2 if the test offered quantitative test results with appropriate upper limits of quantitation, but this warrants further discussion in the context of this type of multi-parametric test result. The complete TPPs are available as supplemental materials that accompany this article, with key sections for use-case #3 discussed below.

### Discussion on select components of use-case #3 TPP—Confirming break in transmission

#### Section 1: Intended use

This diagnostic confirms that transmission of each STH has been sustainably suppressed below its breakpoint, a qualitative population-level test result provided by statistical analysis of pooled specimens or data aggregated from multiple individual-level tests. A negative test result confirms the decision to wind down an active intervention and transition program goals to surveillance for recrudescence (use-case #4). A positive test result indicates that transmission has not been broken, requiring the program to investigate causes of confounding results. An STH program manager would use this test when other metrics indicate that the intervention has likely met its end point and seeks confirmation with a diagnostic survey of a targeted population. These nondiagnostic indicators to initiate testing have yet to be defined and will be clarified through ongoing research assessing the strategy for interrupting transmission [38].

The minimum detection requirement is species-level infection by *A*. *lumbricoides*, *T*. *trichiura*, and hookworms (*A*. *duodenale*, *N*. *americanus*). Hookworm differentiation is not required because interventions are the same for the two species. However, some STH programs or the research community may be interested in differentiation between *A*. *duodenale* and *N*. *americanus* or detection of infections by *A*. *ceylanicum* and *Strongyloides stercoralis*, and these possibilities were listed as optimal criteria. It was noted that neither infections by *A*. *ceylanicum* nor *S*. *stercoralis* are treated by MDA-based interventions [39]. In addition to hookworms, optimal requirements also considered integration of STH programs with those focused on controlling schistosomiasis (*S*. *mansoni*, *S*. *japonicum*, and *S*. *haematobium*) [1, 26].

The intended use of this assay also describes the ideal scenario for implementing a test. These details are described in Section 4 of the TPP and must be realistic to the workflow and resources available to an STH program. These considerations also define criteria for additional methods and accessories required to support the use of the test, such as those for specimen collection and preservation. Health economics and community-acceptability studies that provide a cost-effectiveness and implementation framework for elimination programs are needed to determine the ideal diagnostic scenario.

#### Section 2: Population needs and performance characteristics

The results from a test meeting the needs of use-case #3 provide an indicator of worm and population dynamics to determine if an intervention has reduced parasite reproduction to a point at which local extinction is highly probable (i.e., transmission breakpoint) [40]. In this use-case, criteria for clinical sensitivity is based on an individual’s intensity of infection in relation to this transition point in transmission dynamics, with a true positive test result identifying an individual who is transmitting any STH infection [41]. Phenotypic characteristics related to the number of worms harbored by an individual who would be classified as positive under this use-case remain undefined, as individuals classified as test negative may still be infected with STH but not contributing to transmission. Early-stage research is aimed at developing biomarkers that are fit for this context of use and can be measured in non–stool-based specimens. As with any biomarker-based measurement, it is important to assess the reliability of results by addressing potential sources of interindividual variability, including age, nutritional status, and social dynamics. These variables might be approximated by mathematical and animal modeling to guide biomarker and epidemiological research [40]. The early-stage nature of these investigations is reflected in this version of the TPP and is subject to updates, as additional evidence justifies a rigorous performance requirement.

Analytical sensitivity describes technical performance of the assay (e.g., with spiked samples) and defines the required minimum concentrations of a target analyte that must be measured reliably (95% confidence). Only the lower LOD needs to be defined for a qualitative test and set below the confidence intervals for a diagnostic cutoff. Since this cutoff cannot yet be defined, key opinion leaders agreed that a test must have superior analytical sensitivity compared to current FEC methods. Given the current lack of validated analytical comparators in this range of light infection, in the interim, the LOD was defined as less than 1 egg per 41.7 mg of homogenized stool (equivalent to 24 eggs per gram). This section will be updated in future TPPs as evidence becomes available to justify an appropriate unit of measurement related to clinically relevant diagnostic cutoffs for this use-case, with validated analytical benchmarks that are not egg-based measurements or limited to stool samples.

Quality control requirements address confidence in test results and consider costs for integrating controls within an individual assay as well as costs and resources for external assessments. For stool-based specimens, there was consensus that individual assays require internal controls as a pre-analytical assurance that stool samples were uniformly homogenized and prepared, addressing some of the challenges described in Table 1. There was also agreement that external quality assessment programs would be needed to ensure that STH testing locations that likely vary in infrastructure and workforce are providing consistent results [42, 43].

#### Section 3: Regulatory and statutory needs

The regulatory pathway for global health diagnostics was not defined when the Kato-Katz method was recommended by WHO in 1985 for schistosomiasis control programs [44]. This method would likely not have passed current regulatory requirements if introduced today, given the risk of variable test results and lack of quality control. There was consensus that quality results and reproducibility will be required for any new tests and that these products must be developed using design-control processes [45] and standards defined by ISO13485 [46]. The latter is an internationally recognized standard for developing medical devices through documented processes that ensure consistent attention to quality considerations, from design and development to manufacture and delivery.

In addition to adherence to International Organization for Standardization (ISO) processes, the product-development process will also be defined by the regulatory labeling of the tool for research use only (RUO), investigational use only (IUO), or as an in vitro diagnostic (IVD). Beyond ISO and design control requirements, the regulatory pathway for this assay is currently not known and will be updated in future TPPs.

Tests that guide individual-level treatment decisions are typically classified as IVDs and may also require WHO’s prequalification (PQ) for use in global health settings, in addition to clearance by stringent national regulatory authorities, before they can be procured and used within a program [47]. Unlike traditional IVDs, STH programs do not make individual-level treatment decisions but instead focus on MDA with albendazole or mebendazole. The assay described in use-cases #1 and #3 guide treatment decisions that have population health implications for correct and incorrect results; premature cessation of population-based treatment could result in recrudescence [48]. An alternative consequence is overtreatment and wasted program resources. These types of assays would likely be developed following a regulatory pathway for an IVD, whereas RUO may be suitable for use-cases #2 and #4.

Future discussions are needed to determine the regulatory pathway of tests described in all four use-cases. One important consideration is the implication of a decision based on an incorrect test result and steps to mitigate unintended health outcomes. For use-case #3, an increased risk of recrudescence due to premature wind-down of a program might be mitigated if tests described in use-case #4 are in place and populations remain under surveillance. Likewise, an increased risk of overtreatment due to late wind-down of a program might be mitigated if monitoring tests described in use-case #3 are in place. For use-cases #2 and #4, if the intended purpose of monitoring and surveillance tests is to trigger confirmatory testing or initiate testing to investigate inconsistent results, then these assays would only guide program testing, not treatment decisions. These hypothetical scenarios exemplify the intertwining nature of program guidelines with the regulatory pathway for developing an assay.

#### Section 4: Healthcare/program system needs

This section includes requirements for the successful implementation of a diagnostic. Because targeted population-level data are required for the program decision, minimum data requirements also include geospatial information. There was no advantage for receiving the test result at the point of contact (e.g., rapid diagnostic test) because population-level data is required for program decisions, with acceptable turnaround time for results being over a period of weeks and months.

For the testing environment, key opinion leaders agreed that the testing site should be in proximity to a community or school, preferably at a district-level health center or through a mobile van campaign, to increase community participation and adherence in MDA events. This was based on reports of community involvement in improving the health outcome provided by MDA, particularly because STH infection is predominantly driven by an individual’s interaction with their local environment, including access to clean water and sanitation [49, 50]. The global neglected tropical disease (NTD) agenda is also aligned with aims to strengthen general healthcare services within impoverished communities, increasing opportunities for developing an STH diagnostic on platforms that address other community health needs [51].

District-level settings often have sufficient resources to perform simple diagnostic tests, such as microscopy or rapid diagnostic tests, with access to running water and sufficient electricity during test operation and at least one individual who can be trained to perform a simple test. These settings rarely have sterile work stations and minimal biosafety resources; thus, TPP requirements address the safety of the test operator and local environment by reducing exposure to biospecimens and reagents through design (e.g., self-containment and safety lock) as well as simple disposal processes. Optimal requirements would be met if the test did not require consistent electricity to operate, such as through a battery, and thus were operational in less-resourced settings.

## Conclusion

The success of the current WHO STH control strategy has catalyzed interest in moving beyond coverage estimates and morbidity control to improving program efficiency and exploring the prospect of breaking the transmission of STHs to reduce resources required for sustaining vertical STH programs. These aspirations require surveys of the targeted populations, and the aim of the hypothetical use-cases was to simulate STH program decisions requiring diagnostic information. The context within these use-cases frame performance and implementation requirements for the design and evaluation of existing and new tools. This approach ensures that user needs are the destination of a research and product development road map, instead of forcing the adoption of an imperfect technology. In the best-case scenario, one technology is able meet the requirements of multiple TPPs.

The current TPP for use-case #1 and #2A addresses the needs of morbidity control programs but does not address the needs of elimination programs that would initiate or continue in lower transmission settings (e.g., <20% apparent prevalence). The Kato-Katz method meets most, but not all, of the minimum criteria in this TPP, as it offers sufficient analytical and clinical sensitivity but does not meet precision and reproducibility criteria (§2.5 and 2.6), nor does it meet regulatory requirements (§3.1). New tools that meet all requirements described in this TPP are needed as one step towards strengthening STH program efficiency. Opportunities for improvement are also highlighted in Table 1, and as with any new tool, it is important to consider manufacturability, use, and cost-effectiveness from the program perspective.

STH programs that aim to move beyond morbidity control towards interruption of transmission are described in use-cases #2B, #3, and #4. Tests for use-case #2B will require a lower LOD than use-case #2A, but there is insufficient information to provide definitive criteria, as this approximates the transmission breakpoint. There is a need to define the epidemiological characteristics of a transmission breakpoint to understand the risk criteria of an individual’s contribution to STH transmission within a given population. This is challenging given the wide range of contextual factors that define population heterogeneity, such as seasonality, individual health/nutritional status, environmental exposure, and/or social behaviors. These factors may influence STH transmission and thus, also, breakpoints [52]. Mathematical modeling and animal studies can approximate the extent of these potential contributions to guide definitions of phenotypic characteristics (§2.1), information necessary for defining clinical utility requirements of biomarkers. A TPP for use-case #2B and #3 diagnostics also requires proof of concept that programs can interrupt transmission in a scalable and cost-effective manner, with a strategy that verifies decision points requiring diagnostic surveys and test implementation scenarios (timing, sampling size, etc.).

Diagnostic needs will adjust over time as emerging research continues to evolve program strategies. These TPPs and use-cases are living documents that capture the current trajectory of STH programs to identify gaps that can be addressed through research and product development.

**Key learning points**

- The soil-transmitted helminth (STH) community has started exploring opportunities to strengthen a control program’s ability to monitor changes in prevalence of infection, potentially to a reduction that is sustained below transmission breakpoints.
- Current global strategies have been successful with existing diagnostics, and more ambitious program end points would likely require different tools to evaluate the impact of population-directed interventions.
- Newly created target product profiles (TPPs) described in this article aim to direct the development and evaluation of diagnostic tools that improve the efficiency of control and elimination programs.
- The STH community lacks a tool to confirm a break in transmission, and based on the new TPP, critical evidence to inform the development of this diagnostic is currently unavailable. Additional research is needed to define species-specific transmission breakpoints and guide the translation of individual-level test results to population-level transmission indicators.

## Supporting information

S1 File Overview of diagnostics use-cases for STH control and elimination programs. STH, soil-transmitted helminth.
(PDF)

S2 File Target product profile for STH use-case #1 and #2A diagnostic (mapping, monitoring population-level intervention). STH, soil-transmitted helminth.
(PDF)

S3 File Target product profile for STH use-case #3 diagnostic (confirming decision to stop population-level intervention). STH, soil-transmitted helminth.
(PDF)
