# Supplementary material for: Diagnostic tools for soil-transmitted helminths control and elimination programs: A pathway for diagnostic product development
Source: PLoS Negl Trop Dis. 2018 Mar 1;12(3):e0006213. doi: 10.1371/journal.pntd.0006213 (PMC5832200; doi:10.1371/journal.pntd.0006213)
Supplement: S1 File — STH, soil-transmitted helminth. (PDF) [file pntd.0006213.s001.pdf]

	Use-cases				
	**1. Initiate MDA, determine type of MDA**	**2a. Monitor/Modify MDA**	**2b. Monitor/Modify MDA**	**3. Break in Transmission - Stop MDA**	**4. Post-MDA Surveillance**
**Intended Use**	In high to moderate transmission settings, this *in vitro* assay is for the quantitative detection of eggs as a measure of the presence and intensity of infection by soil-transmitted helminths (STHs, *Ascaris lumbricoides, Trichuris trichuria,* hookworms - *Necator americanus* , and *Ancylcostoma duodenale*) in individual or pooled stool specimens. This assay is intended for use by STH programs to test individuals of any STH infection, as an aid in epidemiological mapping by implementation unit, with ranges of prevalence and infection intensity relevant to the design of STH morbidity control programs.	In high to moderate transmission settings, this *in vitro* assay is for the quantitative detection of eggs as a measure of the presence and intensity of infection by soil-transmitted helminths (STHs, *Ascaris lumbricoides, Trichuris trichuria, Necator americanus* , and *Ancylcostoma duodenale*) in individual or pooled stool specimens. This assay is intended for use in populations undergoing mass drug administration with albendazole or mebendazole. This assay is intended to be used as an aid in monitoring and evaluating the effectiveness of programs aiming to control the morbidity of any STH infection by measuring the impact on prevalence.	This *in vitro* assay is for the quantitative detection of non-microscopy biomarkers as a measure of the presence and intensity of infection by soil-transmitted helminths (STHs, *Ascaris lumbricoides, Trichuris trichuria, Necator americanus* , and *Ancylcostoma duodenale*) in individual or pooled specimens/samples. This assay is intended for use in populations undergoing mass drug administration with albendazole or mebendazole. This assay is intended to be used as an aid in monitoring and evaluating the effectiveness of programs aiming to control the morbidity or interrupt the transmission of STH infection by measuring the impact on prevalence.	This *in vitro* assay is for the qualitative detection of biomarkers as a measure of infection transmission risk by soil-transmitted helminths (STHs, *Ascaris lumbricoides, Trichuris trichuria, Necator americanus* , and *Ancylcostoma duodenale*) in individual or pooled specimens/samples. This assay is intended for use in populations that have received appropriate intervention such as mass drug administration with albendazole or mebendazole. This assay is intended to be used as an aid by STH elimination programs to confirm a break in transmission.	This *in vitro* assay is for the qualitative detection of biomarkers as a measure of exposure by soil-transmitted helminths (STHs, *Ascaris lumbricoides, Trichuris trichuria, Necator americanus* , and *Ancylcostoma duodenale*) in individual or pooled specimens/samples. This assay is intended for use in populations in which mass drug administration and/or other interventations have successfully broken transmission. This assay is intended to be used as an aid by a public health program to confirm sustained break in transmission.
**Prevalence of infection**	High, moderate	High, moderate	High, moderate, low, very-low	Low, very-low	Very-low to zero prevalence
**Populations tested**	At-risk populations	At-risk populations undergoing prescribed intervenation, such as mass treatment with albendazole or mebendazole			Indicator population (eg. 1st graders born after last round of MDA)
**Type of STH program**	Elimination as a public health problem		Elimination as a public health problem and/or interruption of transmission	Interruption of transmission	
**Program decisions from test results**	1. Identify implementation units eligible for mass drug administration (MDA) based on prevalence ranges above/below pre-determined thresholds<br>2. Determine frequency and duration of MDA<br>3. Determine frequency of future testing	1. Determine whether program is on track, based on changes in prevalence from baseline or previous measurement<br>2. If results show that a program is not on track, additional evaluation is needed to guide program modifications		1. Positive result: Confirm decision to stop MDA<br>2. Negative result: Evaluate population for root-cause (eg. therapeutic resistence, poor drug quality, etc)	1. Positive result: Evaluate re-initiation of MDA or other intervention<br>2. Negative result: Verification of sustained break in transmission
**Test results**	1. Quantitative (high-moderate) ranges of infection intensities of each STH species, based on aggregation of individual fecal egg counts		1. Quantitative (high-moderate-low-very low) ranges of infection intensities of each STH species, based on aggregation of individual test results	1. Qualitative (low, very-low) range of infection intensity of each STH species, based on aggregation of individual test results	1. Qualitative (no, very-low) for exposure to any STH, based on aggregation of individual test results
	2. Differentiation between *Ascaris lumbricoides, Trichuris trichiura,* hookworms *(Ancylostoma duodenale* and *Necator americanus)*				
**Type of specimen**	Stool		Stool, blood, urine, saliva		Blood, urine, saliva
**Unit of measurement**	Ranges of fecal egg counts for infection by *Ascaris* spp, *Trichuris* spp, hookworm		Ranges of biomarker abundance		
**Epidemiological/clinical correlation**	Baseline prevalence of high and moderate intensity infection (worm burden)	Impact of MDA on prevalence of high- and moderate- intensity infection (worm burden)		Impact of intervention on breaking transmission (worm burden)	Surveillance for recrudescence (exposure biomarkers)
**Adverse effects of false positive results**	Additional resources for additional testing or unnecessary treatment				
**Adverse effects of false negative results**	Untreated endemic geographies	Incorrect modification of program (eg. Reduced treatment frequency, pre-mature cessation of program)		Increased risk of recrudescence	
**"Nice-to-have" characteristics**	Integration with schistosomiasis control program				Integration with other surveillance programs
**Preferred implementation scenario**	District primary healthcare setting; mobile laboratory				Centralized laboratory
