# Supplementary material for: Diagnostic tools for soil-transmitted helminths control and elimination programs: A pathway for diagnostic product development
Source: PLoS Negl Trop Dis. 2018 Mar 1;12(3):e0006213. doi: 10.1371/journal.pntd.0006213 (PMC5832200; doi:10.1371/journal.pntd.0006213)
Supplement: S2 File — STH, soil-transmitted helminth. (PDF) [file pntd.0006213.s002.pdf]

Diagnostic - Target Product Profile

Disease Area: Soil-Transmitted Helminths – use-case 1 & 2A
Intervention/Candidate: Mass drug administration

Version: 3.1 1-AUG-2017

Companion document: STH diagnostic use-case definitions

Table of Contents

1 INSTRUCTIONS FOR USE 3

2 MEDICAL NEED / DIFFERENTIATION STRATEGY/USE CASE: 4

3 PRODUCT REQUIREMENTS WITH ANNOTATIONS 5

4 CHANGE MANAGEMENT 15

1 Instructions for Use

- The templates are divided into two sections: **1) Executive Summary with Annotations and 2) Additional Variables of Interest**
    - The **Executive Summary with Annotations** captures the minimum and optimistic characteristics for the Intervention or Candidate to be developed.
        - The 1st 3 columns (Executive Summary) can easily be cut and pasted into presentations for discussion.
        - The Annotations column provides the ability to capture the rationale on the thinking and data or references that support the minimum and optimistic targets. It is suggested to capture the annotations during the TPP development dialogue to document the current thinking and data sources.
        - The variables noted with an asterisk(*) are included in the IPDP Executive Summary
    - The **Additional Variables of Interest** are variables which are not relevant to every intervention or candidate. The team should review and assess during TPP development and ongoing reviews.
- The TPP templates capture two sets of characteristics: **Minimum and Optimistic**
    - The **Minimum** criteria are the set of performance and use characteristics to achieve the minimally acceptable level of global health impact (based on modeling, uptake, prevention of cases etc.). These criteria provide context for defining clear go/go no decision criteria that can be applied throughout the development process.
    - The **Optimistic** criteria are the set of performance and use characteristics of an optimistic or ideal product for which the global health impact should be broader, deeper, quicker, etc.
    - Criteria should be quantitative in nature. Subjective language such as “better” or “safer” should be avoided and replaced by quantitative criteria such as “at least X% protection from infection 2 years after initial innoculation” or “no more than Y% Grade 2 adverse events”.
- The **Change Management** section is to capture the changes made to each version of the TPP to enable clear tracking of the evolution of the TPP.
    - Version numbering convention (major/minor as determined by the strategy team)
        - Major version changes should be reflected as V1.0, V2.0, V3.0, etc.
        - Minor version changes should be reflected as V1.1, V1.2, V1.3, etc.
    - Person responsible for maintaining the TPP and making the change should be captured.

2 Medical Need / Differentiation Strategy/Use Case:

Medical Need: Stool-based microscopy is the only tool available to programs aiming to control transmission of infection by soil-transmitted helminths [STHs, *Ascaris lumbricoides, Trichuris trichuria,* and hookworms (*Ancylostoma duodenale* and *Necator americanus*)]. This technique is suitable for geographies with heavy to moderate transmission to measure fecal egg counts for quantifying intensity of infection by *Ascaris* spp*, Trichuris* spp, and hookworms**.** However, the influence of operator-based variability on quality of data are amplified in lower transmission settings.

This *in vitro* assay is for the quantitative and species-specific detection of STH eggs to determine heavy or moderate intensity of infection in individual or pooled specimens. This assay is intended for use in non-treated (use-case #1) or treated (use-case #2) individuals residing in high to moderate prevalence geographies (eg. >20% prevalence, via single measurement by Kato Katz). This assay is intended to be used as an aid in mapping implementation units with ranges of prevalence and infection intensity relevant to the design (use-case #1) and monitoring (use-case #2) of STH morbidity control programs.

Intended Use Case Scenario: This assay is to be used in a mobile or district-level laboratory equipped with dedicated electricity and running water. Stool samples will be transported to the laboratory by field teams. Laboratory technicians will process, analyze, and dispose of the samples at the laboratory. Test results include fecal egg counts for *Ascaris* spp*, Trichuris* spp, and hookworms as an aid in mapping epidemiological implementation units with ranges of prevalence and infection intensity relevant to the design (use-case #1) and monitoring (use-case #2) of STH morbidity control programs.

Critical Assumptions:

1. Measurements of fecal egg counts are suitable for determining individual worm burden of each STH at the high to moderate prevalence settings.
2. Use-cases #1 and #2(A) (mapping and monitoring MDA programs) have requirements that may be addressed by similar technologies/methods.

3 Product Requirements with Annotations

Variable	Minimum<br>*The minimal target should be considered as a potential go/no go decision point.*	Optimistic<br>*The optimistic target should reflect what is needed to achieve broader, deeper, quicker global health impact.*	Annotations<br>*For all parameters, include here the rationale for why this feature is important and/or for the target value.*
**1. Intended Use**			
1.1 What this test shall detect, what is the target organism and/or molecular component to be detected?	Detect and differentiate STHs: *Ascaris* spp, *Trichuris* spp, and hookworms	STHs and *Schistosoma mansoni*	Hookworm species (*N. americanus, A. duodenale)* cannot be distinguished by egg morphology
1.2. Are there variants/genotypes/subtypes to be detected or avoided?	None		
1.3. What is the intended use population for this test?	• Use-case #1: The assay shall be used to test at-risk populations residing in epidemiological implementation units that are suspected or confirmed to have high to moderate prevalence of infection by any STH.<br>• Use-case #2A: The assay shall be used to test at-risk populations to determine a control program's progress against goals, in geographies with high to moderate prevalence of infection by any STH where mass drug administration (MDA) has been initiated.		
1.4. What is the information to be used for? What is the actionable result? Is this an IVD Diagnostics, Screening, or Monitoring Test? Is this a Surveillance or an Investigational Use (IUO) or Research Use Only test (RUO)?	• Use-case #1: The assay shall provide test results that identify implementation units eligible for MDA to control STH transmission, based on prevalence and intensity ranges above / below thresholds defined by WHO<br>• Use-case #1: The assay shall provide test results that determine frequency and duration of MDA to control STH transmission	The assay can provide test results for integrated programs aiming to simultaneously control transmission by both STHs and *S. mansoni*	1. Helminth control in school-age children: A guide for managers of control programmes, Second Ed, World Health Organization

**Variable**	**Minimum**<br>*The minimal target should be considered as a potential go/no go decision point.*	**Optimistic**<br>*The optimistic target should reflect what is needed to achieve broader, deeper, quicker global health impact.*	**Annotations**<br>*For all parameters, include here the rationale for why this feature is important and/or for the target value.*
	• Use-case #1: The assay can provide test results that determine frequency of future testing to inform the use of MDA in controlling STH transmission<br>• Use-case #2A: The assay shall provide test results that determine whether an STH control program is achieving expected results, based on changes from baseline or previous measurement in prevalence of heavy-moderate intensity infection by any STH<br>• Use-case #2A: If test-results demonstrate that a STH control program is not on-track, the suggested intervention would be to initiate additional evaluation(s) to determine whether program modifications are required		
1.5. Is this a qualitative or a quantitative test?	Quantitative, to identify individuals with moderate or heavy intensity of infection by any STH species, relevant to the design and monitoring of STH control programs	Quantitative, to identify individuals with moderate or heavy intensity of infection by *S. mansoni*, relevant to the design and monitoring by an integrated STH and schistosomiasis control program	
1.6. What type of platform/technology is used?	Optical microscope with human based STH differentiation and egg counting	Automated microscope with autonomous STH differentiation and counting	

**2. Individual (Patient) or Population Needs and Performance Characteristics**			
**Variable**	**Minimum**<br>*The minimal target should be considered as a potential go/no go decision point.*	**Optimistic**<br>*The optimistic target should reflect what is needed to achieve broader, deeper, quicker global health impact.*	**Annotations**<br>*For all parameters, include here the rationale for why this feature is important and/or for the target value.*
2.1. Clinical sensitivity	• Heavy-intensity infection: 95% for all STH species (*Ascaris lumbricoides, Trichuris trichiura,* and hookworms)<br>• Moderate-intensity infection: 95% for all species (*Ascaris lumbricoides, Trichuris trichiura,* and hookworms)<br>• Low-intensity infection: equivalent or superior to single Kato Katz-based measurement	*S. mansoni*: 95% for heavy and moderate intensity infections	1. Single Kato-Katz as reference standard<br>2. Eg. University Ghent / STARWORMS SOP 02: Duplicate Kato-Katz smears (Tools>SOPs http:www.starworms.org, accessed 6/28/2017)
2.2. Clinical specificity	• Equal or superior to clinical specificity of single Kato Katz based measurement of individuals with heavy and moderate intensity infection		Reference: Speich B, et. al, *Parasit Vectors*, 2015, 8:82
2.3. Analytical specificity / cross reactivity	• The Assay shall only detect STH species<br>• The Assay shall not cross react with bacteria, yeast, fungi, virus, debris, or non-STH eggs normally or pathologically present in the gastrointestinal tract	The Assay can detect eggs produced by all STH and *S. mansoni*	
2.4. Analytical sensitivity LOD, LOQ, linearity, and quantitative ranges	Limit of detection (LOD) at 95% detection<br>• *A. lumbricoides*: 24 EPG<br>• *T. trichiura*: 24 EPG<br>• Hookworm: 24 EPG	*S. mansoni*<br>• LOD = 24 EPG<br>• ULOQ = 17 absolute egg count	1. EPG = eggs per gram of feces<br>2. Cut-offs based on Kato Katz as comparator

	Limit of quantitation (LOQ) at 95% detection<br>• All species: 24 EPG<br>Upper limit of quantification (ULOQ)<br>• *A. lumbricoides:* 2084 absolute egg count<br>• *T. trichiura:* 417 absolute egg count<br>• *Hookworm:* 167 absolute egg count		method and WHO high-moderate cut-offs
2.5. Precision & reproducibility	• Comparable outcome in 95% of cases.		Reference: Speich B, et. al, *Parasit Vectors*, 2015, 8:82 and Easton *et al*, *Parasites & Vectors* (2017) 10:256
2.6. Internal & external quality control/quality assurance & calibration requirements	• During stool homogenization, an internal control is included as part of quality assurance<br>• Manual calibration with controls pre-loaded or spiked into cartridge<br>• Read-out controls spiked in assay as part of quality assurance of egg visualization	• Self-calibration with extraction and read-out controls pre-loaded in cartridge<br>• Extraction and read-out controls pre-loaded in cartridge	
2.7. Test robustness requirements	• The Assay shall consistently produce > 95% valid results		

2.8. What is the risk of an inaccurate result?	• A false positive result provides a higher apparent prevalence for a given population, increase risk of over-treatment and unnecessary deployment of program resources.<br>• A false negative result provides a lower apparent prevalence for a given population, potentially resulting in under-/non- treatment with increased risk of non-controlled transmission of STH infection.	

**3. Regulatory, Statutory Needs**			
**Variable**	**Minimum**<br>*The minimal target should be considered as a potential go/no go decision point.*	**Optimistic**<br>*The optimistic target should reflect what is needed to achieve broader, deeper, quicker global health impact.*	**Annotations**<br>*For all parameters, include here the rationale for why this feature is important and/or for the target value.*
3.1. What type of global and local regulatory approvals and standards are needed before commercialization in different countries?	• ISO 13485:2003 / ISO 13485:2016		
3.2. What type of promotional, educational, marketing & sales materials are allowed?	• Integrated as part of STH morbidity control program<br>• Training/education: less than 8 hrs	• Integrated as part of STH and schistosomiasis morbidity control program	

**4. Healthcare System Needs**			
**Variable**	**Minimum**<br>*The minimal target should be considered as a potential go/no go decision point.*	**Optimistic**<br>*The optimistic target should reflect what is needed to achieve broader, deeper, quicker global health impact.*	**Annotations**<br>*For all parameters, include here the rationale for why this feature is important and/or for the target value.*
4.1.1 Where the test is to be performed? At what HC level? What are the environmental conditions?	• Mobile or Central Lab at District level<br>• Operating environment suitable for performing other simple diagnostics tests (ie microscopy, rapid diagnostic test, other automated diagnostics)	Remote area, primitive facility	
4.1.2. Workflow requirements. What type of throughput is needed? How fast the result is needed?	Equal or superior to the complete workflow required to perform a single Kato-Katz measurement (specimen to data analysis)	Sample preparation and readout in a single integrated device	Throughput and turnaround times TBD, based on modelling studies evaluating cost-effective diagnostic scenarios
**4.2. Instrument & Device Characteristics**			
4.2.1. Instrumentation physical dimensions; modularity; weight; and level of automation	Bench top instrument	• Mobile instrument: <2kg	
4.2.2. Instrumentation power and water requirements	• Microscope requires running electricity during time of testing<br>• Running water	• Read-out system is battery operated; 24 hr independent operation; computing unit on battery or electricity net	
**4.3. Information & Communication Technology**			
4.3.1. User interface and data input requirements	• Simple / manual operation similar to preparation of Kato Katz slides<br>• Data input includes specimen ID	• Barcode based tracking of specimens and test results<br>• Minimal operator-interference	

4.3.2. Data output, access, security, storage and communication (connectivity) requirements	Test results recorded in paper-based log book.	• GPS location for sample collection<br>• CMOS sensor for data capturing;<br>• Data export for external quality assurance<br>• CMOS sensor captures picture of fecal egg count;<br>• Data display: picture and interpretation table	
4.3.3. How are the results transmitted?	• Test results manually logged into paper-based logbooks or in a computer based database	• Wireless transfer of specimen ID and test results to computing unit<br>• Analysis by egg counting software<br>• Egg count picture and analysis uploaded on cloud server (such as DHIS2, or intermediary server)	DHIS2 = District Health Information System version 2 https://www.dhis2.org/overview
**4.4 Reagent and control handling**			
4.4.1. How shall the reagents/cartridges be stored? How shall they be packaged?	• The assay reagents/cartridges and controls shall demonstrate stability up to one year at 40°C<br>• Operational up to 40°C	• The assay reagents / cartridges and controls shall demonstrate stability up to two years at 40°C	Minimal shelf-life to be modelled.
4.4.2 Waste management & biosafety requirements	• Sample preparation will not require biosafety controlled environment		

	beyond personal protective equipment (PPE)<br>• Once specimen is deposited inside cartridge, system is closed and contained from outside environment with a safety lock<br>• SOP provided for cleaning materials used and workspace<br>• Cartridge and sample prep material disposal in biosafety waste bin following standard (WHO and country) medical waste guidelines<br>• Remaining biospecimens and unused samples in latrine/toilet (for non-blood samples)		
**4.5. Sample Handling**			
4.5.1. What type of specimens and assays are to be run in the same facility? How are the specimens received?	Individual stool samples collected within 8 hours in standard stool collection containers.	The assay can test preserved stool specimens transported according to collection device labeling.	
4.5.2. Sample type(s) and volumes	Less than 30mL of homogenized stool		
4.5.3. Sample collection & processing requirements	• Stool specimens collected by field officer within a community or school-based campaign<br>• Individuals are provided with standard stool container, spatula/tounge depressor, mat for deposition, and toilet paper. Individuals provide stool specimen and place an aliquot inside container.		
4.5.4. Sample preparation requirements	• The assay requires a limited amount (<3) of simple steps.	• Sample in – results out (eg. Homogenize stool in standard container, add into	Extra material is required for repetition of test in case of failure, or for use by other assays

	• The assay shall require 42 mg as a minimum amount of stool, but no more than ¼ of the total amount of specimen collected	cartridge, all sample extraction procedures performed on cartridge)	
**4.6. Distribution, Service & Support, Training**			
4.6.1. Who will run the test? How he/she will be trained and supported?	• The Assay shall run on equipment by a skilled worker available at the level of the facility	• The Assay shall run on an easy-to-use automated system by a lay person (education level and functional literacy equivalent to 8th grade US)	
4.6.2. What type of Quality Control System is needed to monitor test/site performance on ongoing bases? What other support is needed?	• Internal quality control for sample preparation and analysis (eg. spiked beads, see §2.6)<br>• Quality Controls by proficiency panels offered as part of a manufacturer sponsored QA program<br>• Quality Control can be performed using equipment and skilled personnel available at the level of the facility	• Quality controls and connectivity system to remotely monitor assay, instrument performance, and operator proficiency, should be offered as part of a manufacturer sponsored QA program	
4.6.3. Instrument & test supply reliability	• The Assay can be maintained by a skilled worker with equipment available at the level of the facility, with minimal external support	• Remote monitoring and diagnosis<br>• Preventive maintainance: no more than three per year at launch and one after two years<br>• In-country service engineers	
4.6.4. Service & support response time	Manufacturer shall provide technical support to address assay and instrument issues and customer complaints	Manufacturer should replace and return non-functioning units	

**5. Commercial and Sustainability Needs**			
**Variable**	**Minimum**<br>*The minimal target should be considered as a potential go/no go decision point.*	**Optimistic**<br>*The optimistic target should reflect what is needed to achieve broader, deeper, quicker global health impact.*	**Annotations**<br>*For all parameters, include here the rationale for why this feature is important and/or for the target value.*
5.1. In what countries will be launched? Is controlling the disease a priority for the government of the countries we intend to supply? Are their timelines in alignment with ours? Is advocacy needed?	All endemic countries with a planned or existing national STH program		
5.2. What are the funding agencies that would support it? Are there any IP global access issues? Who would negotiate them?	TBD		
5.3. What are commercial channels?	STH programs as end-user; commercial channels TBD		
5.4. What does the total end-user price per test (reagents & consumables; ex-works) need to be? What is the maximum cost of the instrumentation (per module as applicable)?	Target price based on value of improved efficiency and effectiveness of STH control program, compared to current use of Kato-Katz		• Reference: HC Turner et al, Trends in Parasitology, 2017, 33(6), 435-443.<br>• Cost-effectiveness to be modeled for post TPPv3.1.

4 Change Management

Version	Key Changes from previous version	Change Made By
V1.2<br>3 May 2017	Initial draft	Mark Lim
V1.3<br>8 May 2017	Modified draft based on comments from internal reviews. Distributed to meeting participants	Mark Lim
V2.0<br>8 June 2017	Draft based on pre-workshop discussion. Distributed to workshop participants	Mark Lim
V3.0<br>28 June 2017	Multiple changes based on consensus arrived at Annecy 2017 workshop	Mark Lim
V3.1<br>1 Aug 2017	TPP finalized, version locked as V3.1	Mark Lim

Template Change Management

Version	Key Changes from previous version	Change Made By
V 2.0<br>17 JULY 2015	• Created based on Diagnostics Update July 14 2015	Janet White
