# Supplementary material for: Diagnostic tools for soil-transmitted helminths control and elimination programs: A pathway for diagnostic product development
Source: PLoS Negl Trop Dis. 2018 Mar 1;12(3):e0006213. doi: 10.1371/journal.pntd.0006213 (PMC5832200; doi:10.1371/journal.pntd.0006213)
Supplement: S3 File — STH, soil-transmitted helminth. (PDF) [file pntd.0006213.s003.pdf]

Diagnostic - Target Product Profile

Disease Area: Soil Transmitted Helminths – Use case #3
Intervention/Candidate: Mass Drug Adminstration

Version: **V3.1 1-AUG-2017**

Companion document: STH diagnostic use-case definitions

Table of Contents

1 INSTRUCTIONS FOR USE 3

2 MEDICAL NEED / DIFFERENTIATION STRATEGY/USE CASE: 4

3 PRODUCT REQUIREMENTS WITH ANNOTATIONS 5

4 CHANGE MANAGEMENT 16

1 Instructions for Use

- The templates are divided into two sections: **1) Executive Summary with Annotations and 2) Additional Variables of Interest**
  - The **Executive Summary with Annotations** captures the minimum and optimistic characteristics for the Intervention or Candidate to be developed.
    - The 1st 3 columns (Executive Summary) can easily be cut and pasted into presentations for discussion.
    - The Annotations column provides the ability to capture the rationale on the thinking and data or references that support the minimum and optimistic targets. It is suggested to capture the annotations during the TPP development dialogue to document the current thinking and data sources.
    - The variables noted with an asterisk(*) are included in the IPDP Executive Summary
  - The **Additional Variables of Interest** are variables which are not relevant to every intervention or candidate. The team should review and assess during TPP development and ongoing reviews.
- The TPP templates capture two sets of characteristics: **Minimum and Optimistic**
  - The **Minimum** criteria are the set of performance and use characteristics to achieve the minimally acceptable level of global health impact (based on modeling, uptake, prevention of cases etc.). These criteria provide context for defining clear go/go no decision criteria that can be applied throughout the development process.
  - The **Optimistic** criteria are the set of performance and use characteristics of an optimistic or ideal product for which the global health impact should be broader, deeper, quicker, etc.
  - Criteria should be quantitative in nature. Subjective language such as “better” or “safer” should be avoided and replaced by quantitative criteria such as “at least X% protection from infection 2 years after initial innoculation” or “no more than Y% Grade 2 adverse events”.
- The **Change Management** section is to capture the changes made to each version of the TPP to enable clear tracking of the evolution of the TPP.
  - Version numbering convention (major/minor as determined by the strategy team)
    - Major version changes should be reflected as V1.0, V2.0, V3.0, etc.
    - Minor version changes should be reflected as V1.1, V1.2, V1.3, etc.
  - Person responsible for maintaining the TPP and making the change should be captured.

2 Medical Need / Differentiation Strategy / Use Case:

Medical Need:

Stool-based microscopy is the only tool available to programs aiming to control transmission of infection by soil transmitted helminths (STH: *Ascaris lumbricoides, Trichuris trichiura,* and hookworms - *Necator americanus, Ancylcostoma duodenale*). This technique measures species-specific egg counts and is suitable for geographies with significant prevalence. In lighter transmission settings, such as those expected in an effective STH elimination program, the influence of poor sensitivity and operator variability are amplified, negatively impacting data reliability when using this method to determine effectiveness of any prescribed intervention [eg. mass drug administration (MDA)]. Measurements of microscopy-based biomarkers, such as fecal egg counts, are also inappropriate indicators of worm burden at the lighter intensities of STH infection expected in low to very-low prevalence settings.

This *in vitro* assay is for the qualitative detection of non-microscopy biomarkers to determine risk of STH infection transmission in individual or pooled specimens/samples. This assay is intended for use in populations residing in low prevalence settings of any STH infection that have received appropriate intervention such as MDA with albendazole or mebendazole and/or WSH (water, sanitation, hygiene). This assay is intended to be used as an aid by STH programs to confirm a break in transmission.

Intended Use Case Scenario:

This assay is to be used in a mobile or district-level laboratory equipped with dedicated electricity and running water. Simply collected biospecimens will be transported to the laboratory by field teams. Laboratory technicians will process, analyze, and dispose of the samples at the laboratory. Test results will determine if the intervention(s) used by an soil transmitted helminth (STH) elimination program were successful in breaking transmission of infection by each STH (*Ascaris lumbricoides, Trichuris trichiura,* and hookworms - *Necator americanus, Ancylcostoma duodenale*).

Critical Assumptions:

1. The assay described in this TPP will provide a test result that determines if STH transmission within a defined population has or has not been interrupted by intervention(s) prescribed by an STH elimination program.
2. Microscopy-based measurements, such as Fecal Egg Counts, are not appropriate indicators for worm burden at the lower intensities of infection, and thus cannot be used to determine if transmission has been interrupted by prescribed intervention(s).

3 Product Requirements with Annotations

Variable	Minimum<br>*The minimal target should be considered as a potential go/no go decision point.*	Optimistic<br>*The optimistic target should reflect what is needed to achieve broader, deeper, quicker global health impact.*	Annotations<br>*For all parameters, include here the rationale for why this feature is important and/or for the target value.*
**1. Intended Use**			
1.1 What this test shall detect, what is the target organism and/or molecular component to be detected?	The assay must detect at a limit of detection relevant to a break in transmission for all species of soil transmitted helminth (STH): *Ascaris lumbricoides, Trichuris trichiura,* and hookworm (*Ancylostoma duodenale, Necator americanus)*	• The assay can differentiate and detect at a limit of detection relevant to a break in transmission of each hookworm species, *Necator americanus*, *Ancylostoma duodenale and Ancylostoma ceylanicum.*<br>• The assay can detect at a limit of detection relevant to a break in transmission of *Schistosoma mansoni, Schistosoma haematobium, Strongyloides stercoralis.*	• Species-specific prevalence threshold relevant to interrupted transmission to be defined/modelled.
1.2. Are there variants/genotypes/subtypes to be detected or avoided?	None		
1.3. What is the intended use population for this test?	• The assay shall be used to test all populations residing in low to very-low STH transmission settings.<br>• The assay shall be used to test populations residing in epidemiological implementation units that have completed a prescribed intervention,		

Variable	Minimum<br>*The minimal target should be considered as a potential go/no go decision point.*	Optimistic<br>*The optimistic target should reflect what is needed to achieve broader, deeper, quicker global health impact.*	Annotations<br>*For all parameters, include here the rationale for why this feature is important and/or for the target value.*
	such as mass drug administration (MDA) of albendazole or mebendazole and/or WASH (water, sanitation, hygiene) measures.<br>• The assay shall be used when there is sufficient evidence that a prescribed intervention can be stopped.		
1.4. What is the information to be used for? What is the actionable result? Is this an IVD Diagnostics, Screening, or Monitoring Test? Is this a Surveillance or an Investigational Use (IUO) or Research Use Only test (RUO)?	• The assay shall provide test results that, in combination with individual test results obtained from the same pre-defined population, provides an approximation of transmission intensity for use by an STH elimination program.<br>• Test results confirm whether a prescribed intervention such as MDA can be ceased, or in the event of discordant results, an evaluation of program strategy should be initiated.<br>• A test result will indicate that an individual has a minimal to non-existant risk of contributing to transmission, or, an intensity of STH infection related to increased risk of transmission.	• The assay can provide similar qualitative results for schistosomiasis elimination programs.<br>• The assay can provide test results that differentiate between the hookworm species, *Necator americanus* and *Ancylcostoma duodenale*.<br>• The assay can provide test results for programs targeting *Strongyloides stercoralis.*<br>• The assay can provide test results for programs targeting *Ancylostoma ceylanicum.*<br>• The assay is compatible with pooled samples or specimens.	• Quantitative cut-offs relevant to the interruption of transmission by each STH to be approximated by mathematical modelling.<br>• Discordant results could indicate incomplete treatment, poor drug quality, drug resistant worms, non-adherence, etc., and would need to be investigated with another test or assessment.

Variable	Minimum<br>*The minimal target should be considered as a potential go/no go decision point.*	Optimistic<br>*The optimistic target should reflect what is needed to achieve broader, deeper, quicker global health impact.*	Annotations<br>*For all parameters, include here the rationale for why this feature is important and/or for the target value.*
1.5. Is this a qualitative or a quantitative test?	Qualitative	The assay can provide quantitative results related to the intensity of infection by any STH.	Limits of detection, related to worm burden and interruption of transmission, to be informed by mathematical modelling
1.6. What type of platform/technology is used?	Non-microscopy platform with integrated specimen-to-results capabilities.		Technology TBD, informed by cost-effectiveness modelling and feasibility assessments

2. Individual (Patient) or Population Needs and Performance Characteristics			
**Variable**	**Minimum**<br>*The minimal target should be considered as a potential go/no go decision point.*	**Optimistic**<br>*The optimistic target should reflect what is needed to achieve broader, deeper, quicker global health impact.*	**Annotations**<br>*For all parameters, include here the rationale for why this feature is important and/or for the target value.*
2.1. Clinical sensitivity	The assay shall be more sensitive than microscopy-based tests to detect patent infection and/or exposure to recent infection at the population level		Association between individual worm burden and interruption of transmission related to the impact of a program stopping decision to be modeled
2.2. Clinical specificity	Equal or superior to clinical specificity of pPCR based measurements		Reference: Easton AV, *et. al*, *Parasit Vectors*, 2016, 9:38
2.3. Analytical specificity / cross reactivity	• The assay shall not cross react with bacteria, yeast, fungi, virus, debris, or other helminth species normally or pathologically present in the gastrointestinal tract	• The Assay can differentiate between two hookworm species, *Necator americanus* and *Ancylcostoma duodenale*<br>• The Assay can detect infections by *Schistosoma*	

		mansoni, Schistosoma haematobium, Ancylostoma ceylanicum and Strongyloides stercoralis	
2.4. Analytical sensitivity	Limit of detection (LOD) at 95% detection<br>• *A. lumbricoides*: < 24 EPG<br>• *T. trichiura*: < 24 EPG<br>• Hookworm: < 24 EPG		• Current LOD thresholds based on current landscape of fecal egg counting methods.<br>• Thresholds to be updated post TPP v3.1, after mathematical modelling studies inform ranges of detection relevant to worm burden and efficiency of transmission, with comparator / composite platforms.
2.5. Precision & reproducibility	• The Assay shall produce reproducible results (>95% agreement) when the same sample is run across different		Ref: Easton *et al*, *Parasites & Vectors* (2017) 10:256

	instruments, reagent lots, days, settings by different operators.<br>• When the same sample is run across different instruments, reagent lots, days, settings by different operators it should produce results with a %CV <10%.		
2.6. Internal & external quality control/quality assurance & calibration requirements	• The assay shall include an internal control, and negative and positive external controls to confirm the validity and accuracy of the results.		
2.7. Test robustness requirements	• Clinical and analytical performance of the assay should not be affected (i.e. no statistically significant difference in % specificity and/or sensitivity) when potentially interfering exogenous and endogenous substances are present in the sample (preservatives, urine, whole blood, etc.)<br>• The cross-contamination rate for the Assay shall be ≤ 1.0% when high titer specimens are processed along with negative specimens<br>• The Assay shall consistently produce > 95% valid results		High-titer specimens currently > 24 EPG for all species, based on current LOD (§2.4). To be updated after modelling studies post TPP v3.1
2.8. What is the risk of an inaccurate result?	• A false negative result provides a lower apparent prevalence of STH infection for a given population, potentially increasing risk of STH infection resurgence from pre-mature cessation of intervention.		Cost/resource implications to be modeled

	• A false positive result provides a higher apparent prevalence of STH infection for a given population, potentially resulting in unnecessary deployment of progam resources for continued intervention.	

**3. Regulatory, Statutory Needs**			
**Variable**	**Minimum**<br>*The minimal target should be considered as a potential go/no go decision point.*	**Optimistic**<br>*The optimistic target should reflect what is needed to achieve broader, deeper, quicker global health impact.*	**Annotations**<br>*For all parameters, include here the rationale for why this feature is important and/or for the target value.*
3.1. What type of global and local regulatory approvals and standards are needed before commercialization in different countries?	• ISO 13485:2003 / ISO 13485:2016		
3.2. What type of promotional, educational, marketing & sales materials are allowed?	• Integrated as part of STH program<br>• Training/education: less than 8 hrs	• Integrated as part of integrated program simultaneously targeting schistosomiasis.	

4. Healthcare System Needs			
**Variable**	**Minimum**<br>*The minimal target should be considered as a potential go/no go decision point.*	**Optimistic**<br>*The optimistic target should reflect what is needed to achieve broader, deeper, quicker global health impact.*	**Annotations**<br>*For all parameters, include here the rationale for why this feature is important and/or for the target value.*
4.1.1 Where the test is to be performed? At what HC level? What are the environmental conditions?	• Mobile or Central Lab at District level<br>• Operating environment suitable for performing other simple diagnostics tests (ie microscopy, rapid diagnostic test, other automated platforms)	• Remote area, primitive facility	
4.1.2. Workflow requirements. What type of throughput is needed? How fast the result is needed?	Sample preparation and readout in a single integrated device	Pooled samples prepared and readout in single integrated device	Throughput and turnaround times TBD, based on modelling studies evaluating cost-effective diagnostic scenarios
**4.2. Instrument & Device Characteristics**			
4.2.1. Instrumentation physical dimensions; modularity; weight; and level of automation	Bench top instrument	Mobile instrument: <2kg	
4.2.2. Instrumentation power and water requirements	• Running water and consistent electricity during test operation		
**4.3. Information & Communication Technology**			
4.3.1. User interface and data input requirements	• Data input includes specimen ID<br>• GPS location for sample collection<br>• Barcode based tracking of specimens and test results<br>• Minimal operator-intervention		

4.3.2. Data output, access, security, storage and communication (connectivity) requirements	• Barcode based tracking of specimens and test results<br>• Data display: picture and interpretation table	• Prevalence values uploaded on cloud server (such as DHIS2, or intermediary server)	DHIS2 = District Health Information System version 2 https://www.dhis2.org/overview
4.3.3. How are the results transmitted?	• Wireless transfer of specimen ID, GPS location of community, and test results to local computing unit equipped with software to provide population-level data<br>• Test results stored on-board for download to computer via wired or wireless connection	• Test results directly transmitted to national STH program's cloud infrastructure (such as DHIS2, or intermediary server)	
**4.4 Reagent and control handling**			
4.4.1. How shall the reagents/cartridges be stored? How shall they be packaged?	• The assay reagents/cartridges and controls shall demonstrate stability up to one year at 40°C<br>• Operational up to 40°C	• The assay reagents/cartridges and controls shall demonstrate stability up to two years at 40°C	Minimal shelf-life to be modelled.
4.4.2 Waste management & biosafety requirements	• Sample preparation will not require biosafety controlled environment beyond personal protective equipment (PPE)<br>• Once specimen is deposited inside cartridge, system is closed and contained from outside environment with a safety lock<br>• SOP provided for cleaning materials used and workspace<br>• Cartridge and sample prep material disposal in biosafety waste bin		

	following standard (WHO and country) medical waste guidelines<br>• Remaining biospecimens and unused samples in latrine/toilet (for non-blood samples)		
**4.5. Sample Handling**			
4.5.1. What type of specimens and assays are to be run in the same facility? How are the specimens received?	• The assay can test samples from specimens prepared remotely and transported according to collection device labeling.	• The assay can test biospecimens pooled remotely and transported according to collection device labeling.<br>• The assay can test preserved biospecimens, prepared and transported according to collection device labeling.	• Determination of pooling sizes dependent on biomarker, specimen, and statistical models.
4.5.2. Sample type(s) and volumes	• The assay can test samples prepared from easily collected biospecimens, such as stool, blood, urine, or saliva		Total specimen volume dependent on type of specimen and LOD requirements, also see §4.5.4
4.5.3. Sample collection & processing requirements	• Easily collected specimens collected by field officer within a community or school-based campaign	• Specimens can be collected and pooled remotely, and transported according to collection device labeling.<br>• Specimens can be collected and transported according to collection device labeling, and pooled at the testing facility prior to testing (statistical pooling TBD)	• Determination of handling conditions for various specimens based on collection device validation studies
4.5.4. Sample preparation requirements	• The assay shall require no more than ¼ of the total amount of specimen / pooled specimen collected		Extra material for repetition of test, in case of failure or for use by other assays

4.6. Distribution, Service & Support, Training			
4.6.1. Who will run the test? How he/she will be trained and supported?	• The Assay shall run on equipment by a skilled worker available at the level of the facility	• The Assay shall run on an easy-to-use automated system, operated by a lay person (education level and functional literacy equivalent to 8th grade US)	
4.6.2. What type of Quality Control System is needed to monitor test/site performance on ongoing bases? What other support is needed?	• Quality controls and connectivity system to remotely monitor assay, instrument performance, and operator proficiency, should be offered as part of a manufacturer sponsored QA program		
4.6.3. Instrument & test supply reliability	• The Assay can be maintained by a skilled worker with equipment available at the level of the facility, with minimal external support	• Remote monitoring and diagnosis<br>• Preventive maintainance: no more than three per year at launch and one after two years<br>• In-country service engineers	
4.6.4. Service & support response time	• Manufacturer shall provide technical support to address assay and instrument issues and customer complaints	• Manufacturer should replace and return non-functioning units	

5. Commercial and Sustainability Needs			
**Variable**	**Minimum** *The minimal target should be considered as a potential go/no go decision point.*	**Optimistic** *The optimistic target should reflect what is needed to achieve broader, deeper, quicker global health impact.*	**Annotations** *For all parameters, include here the rationale for why this feature is important and/or for the target value.*
5.1. In what countries will be launched? Is controlling the disease a priority for the government of the countries we intend to supply? Are their timelines in alignment with ours? Is advocacy needed?	• All endemic countries with a planned or existing national STH program		
5.2. What are the funding agencies that would support it? Are there any IP global access issues? Who would negotiate them?	TBD		
5.3. What are commercial channels?	STH programs are targeted end-user, commercial channels TBD		
5.4. What does the total end-user price per test (reagents & consumables; ex-works) need to be? What is the maximum cost of the instrumentation (per module as applicable)?	• Target price based on value of diagnostic test result to efficiency and effectiveness of STH elimination program, to be informed by health economics study		Reference: HC Turner et al, Trends in Parasitology, 2017, 33(6), 435-443.

4 Change Management

Version	Key Changes from previous version	Change Made By
V1.0<br>13-MAR-2017	First draft of TPP based on Simon Brooker – Mark Lim discussions	Mark Lim
V1.1<br>08 May 2017	Draft based on internal comments, distributed to workshop participants for additional review	Mark Lim
V2.0<br>05 June 2017	Draft based on pre-workshop discussions, for distribution/comment at Annecy 2017 meeting	Mark Lim
V3.0<br>30 June 2017	Multiple changes based on consensus arrived at Annecy 2017 workshop	Mark Lim
V3.1<br>1 Aug 2017	TPP finalized, version locked as V3.1	Mark Lim

Template Change Management

Version	Key Changes from previous version	Change Made By
V 2.0<br>17 JULY 2015	• Created based on Diagnostics Update July 14 2015	Janet White
